# Modulation of all-trans retinoic acid-induced MiRNA expression in neoplastic cell lines: a systematic review

**DOI:** 10.1186/s12885-019-6081-7

**Published:** 2019-08-30

**Authors:** Lara Lima, Thaísa Cristina Tavares de Melo, Diego Marques, Jéssica Nayara Góes de Araújo, Isabela Samária Fernandes Leite, Camila Xavier Alves, Julieta Genre, Vivian Nogueira Silbiger

**Affiliations:** 1 Postgraduate Program in Nutrition, Federal University of Rio Grande do Norte, Natal, Brazil; 2 Laboratory of Bioanalysis and Molecular Biotechnology, Federal University of Rio Grande do Norte, Natal, Brazil; 3 Postgraduate Program in Pharmaceutical Sciences, Federal University of Rio Grande do Norte, Natal, Brazil; 4 Department of Clinical and Toxicological Analysis, Federal University of Rio Grande do Norte, Av. General Gustavo Cordeiro de Faria S/N, Petrópolis, Natal - RN 59012-570 Brazil

**Keywords:** Cancer, All-trans retinoic acid, miRNA, Expression modulation

## Abstract

**Background:**

Cancer is a genetic and epigenetic disease that involves inactivation of tumor suppressor genes and activation of proto-oncogenes. All-trans retinoic acid (ATRA) is an isomer of retinoic acid involved in the onset of differentiation and apoptosis of a number of normal and cancer cells, functioning as an anti-cancer agent in several neoplasms. Ectopic changes in the expression of certain microRNAs (miRNAs) occur in response to ATRA, leading to phenotypic alterations in neoplastic cell lines. Moreover, the modulation of miRNA patterns upon ATRA-treatment may represent an effective chemopreventive and anti-cancer therapy strategy. The present systematic review was performed to provide an overview of the modulation of ATRA-induced miRNA expression in different types of neoplastic cells and identify the efficacy of intervention factors (i.e., concentration and duration of treatment) and how they influence expression profiles of oncogenesis-targeting miRNAs.

**Methods:**

A systematic search was conducted according to the PRISMA statement via the US National Library of Medicine MEDLINE/PubMed bibliographic search engine.

**Results:**

The search identified 31 experimental studies involving human cell lines from nine different cancer types (neuroblastoma, acute myeloid leukemia, breast cancer, lung cancer, pancreatic cancer, glioma, glioblastoma, embryonal carcinoma, and colorectal cancer) treated with ATRA at concentrations ranging from 10^− 3^ μmol/L to 10^2^ μmol mol/L for 24 h to 21 days.

**Conclusion:**

The concentrations used and the duration of treatment of cancer cells with ATRA varied widely. The presence of ATRA in the culture medium of cancer cells was able to modulate the expression of more than 300 miRNAs, and inhibit invasive behavior and deregulated growth of cancer cells, resulting in total tumor remission in some cases. ATRA may thus be broadly effective for neoplasm treatment and prevention, although these studies may not accurately represent in vivo conditions. Additional studies are required to elucidate ATRA-induced miRNA modulation during neoplasm treatment.

## Background

Cancer is the third leading cause of death worldwide, representing a considerable public health burden [1]. It is anticipated that the impact of this disease will correspond to approximately 26 million new cancer cases per year by 2030 [1]. The initiation and progression of cancer is mainly driven by genetic and epigenetic alterations in DNA and histones that result in the inactivation of tumor suppressor genes or activation of proto-oncogenes [2].

Retinoic acid (RA) is the major bioactive metabolite of retinol or vitamin A and serves as a potent regulator of cell growth, differentiation, and matrix formation of various cell types during embryogenesis [3, 4]. All-trans retinoic acid (ATRA) is an isomer of RA that exhibits dose-dependent effects on differentiation and apoptosis of a number of normal and cancer cells [5]. ATRA has also been shown to function as an anti-cancer agent in several neoplasms, such as gastric cancer [6], breast cancer [7, 8] leukemia [9–12], nephroblastoma [13], melanoma [14], lung cancer [15], and neuroblastoma [16, 17].

miRNAs are endogenous, small, non-coding RNAs that regulate gene expression by binding to their target mRNAs, leading to degradation and/or translational repression [5]. These molecules have been extensively associated with cancer development, as they play important roles in regulating biological processes such as differentiation, cell proliferation, apoptosis, epithelial-mesenchymal transition, cancer metastasis, and angiogenesis [18]. miRNAs can function as oncogenes or tumor suppressors, and their abnormal expression has already been identified in both solid and hematopoietic tumors. Thus, miRNA profiling is a promising strategy for cancer diagnosis and prognosis [19].

Several studies have shown that ectopic expression of certain miRNAs, which are upregulated in response to ATRA treatment, is sufficient to produce phenotypic changes that are typically induced by ATRA [20, 21]. Furthermore, the potential role of miRNAs induced by ATRA in modulating cancer cells has been demonstrated in different cellular contexts [22–29]. Thus, the aim of our systematic review is to provide an overview of the modulation of ATRA-induced miRNA expression in different types of human cancer.

## Methods

This review was written in accordance with the PRISMA statement [30].

### Search strategy

A systematic search of the published literature form January 1, 2007 to November 12, 2018 was undertaken using the US National Library of Medicine MEDLINE/PubMed (www.ncbi.nlm.nih.gov/pubmed) bibliographic search engine. Multiple PubMed searches were conducted using the following keyword combinations “(miRNA OR microRNA) AND (“retinoic acid” OR “vitamin A” OR retinol) AND (cancer OR neoplasm OR tumor).” The studies were then compiled into a single database and duplicates removed. An initial screening was performed by assessing the title and abstract. After reading the selected studies, the references section of each text was analyzed for additional relevant studies.

### Study selection

For this review, we selected studies written in the English language that evaluated changes in the expression profile of miRNAs in neoplastic human cell lines after treatment with ATRA. The exclusion criteria were other systematic reviews on the subject, bioinformatics analyses, short communications, and supporting information.

### Data extraction

Data extraction was conducted by one reviewer (LL) and verified by the other authors. Extracted data included cancer type, cell type analyzed, conditions of ATRA treatment (dose and duration), miRNAs evaluated, and significant findings. For studies that determined ATRA-induced miRNA expression at multiple time points, the values obtained on the last day were considered. All studies are grouped according to the type of cancer.

## Results

**Fig. 1:**
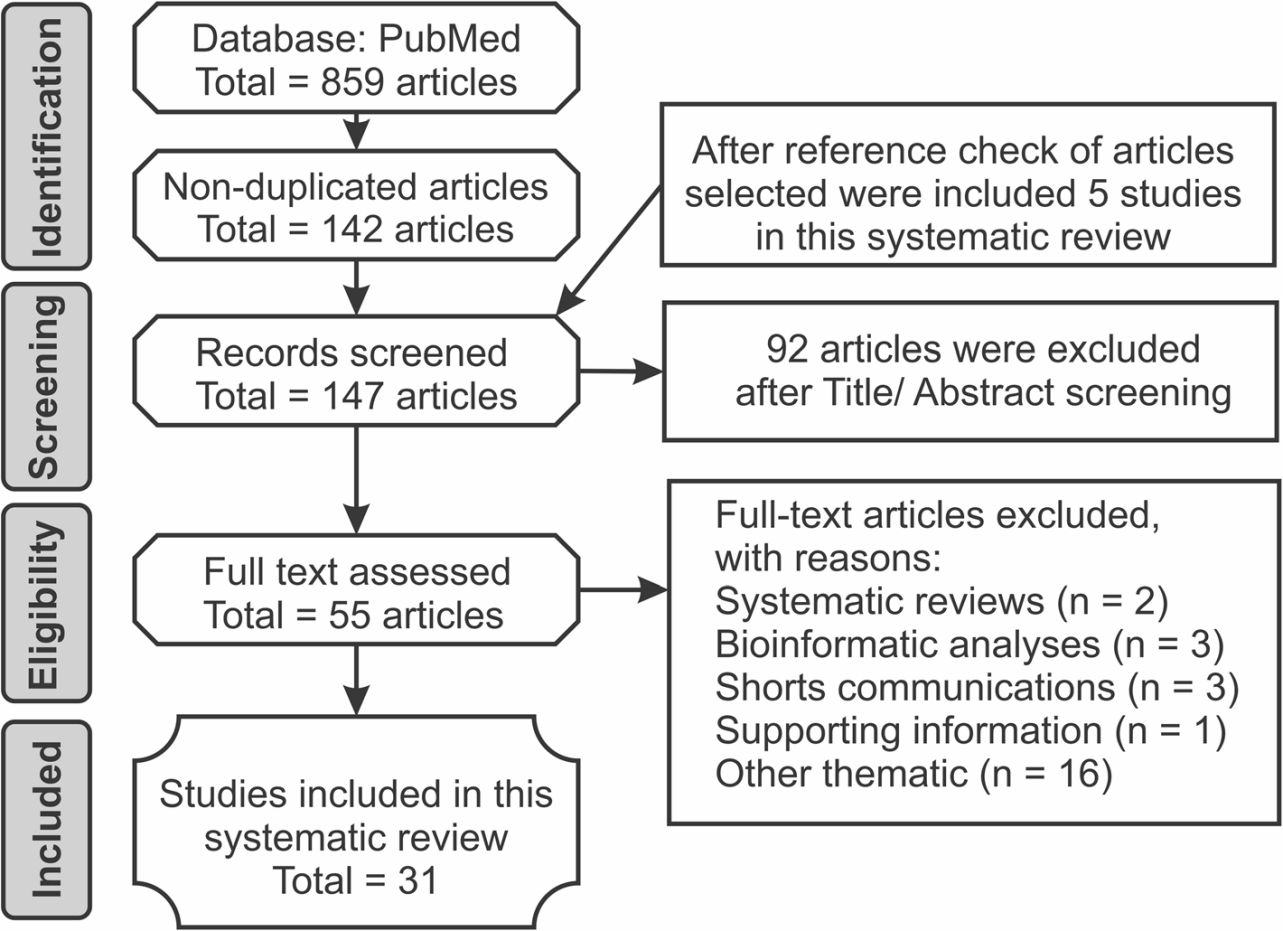
Flow of information through the different phases of the systematic review regarding the modulation of ATRA-induced expression of miRNAs in different types of neoplastic cells

After conducting searches using 18 combinations of key terms, we identified 859 studies. A total of 147 studies were identified through database searching. After screening the abstracts, 92 studies were excluded, and 55 full-text studies were accessed and assessed for eligibility. Reasons for study exclusion included studies describing ATRA-mediated effects not related to miRNA expression or mentioning genes that exhibited modifications induced by ATRA without describing the miRNA involved, systematic reviews, bioinformatic analysis, short communications, and supporting information (Fig. 1).

The characteristics and findings of the 31 studies included in this systematic review are summarized in Tables 1, 2, 3, 4 and 5 according to cancer type. In particular, nine different types of cancer were addressed, neuroblastoma (*n* = 12), acute myeloid leukemia (*n* = 9), breast carcinoma (*n* = 3), lung cancer (*n* = 2), pancreatic cancer (*n* = 1), glioma (*n* = 1), glioblastoma (*n* = 1), embryonal carcinoma (*n* = 1), and colorectal cancer (*n* = 1). All studies were based on human neoplastic cell lines treated with ATRA, followed by evaluation of changes in miRNA expression patterns. One study utilized human and animal cell lines [32]; however, only results related to human cells were included in this review. The results have been grouped according to cancer type and are presented below.

**Table 1:** miRNAs regulated by ATRA in NB cell lines

Study (reference and country)	Cell type	ATRA treatment	miRNAs upregulated	miRNAs downregulated
Chen and Stallings (2007) [31] United States	SK-N-BE	5 μmol/L for 5 days	17; *miR-330, miR-187, miR-331, miR-200c, miR-216, miR-150, miR-141, miR-200a, miR-326, miR-186, miR-30b, miR-137, miR-184, miR-129, let-7b, let-7a* and *miR-181a*; most significant: *miR-184*	4; *miR-323*, *miR-302a*, *miR-181b* and *miR-92*; most significant *miR-192*
Laneve et al. (2007) [20] Italy	SK-N-BE	10 μmol/L for 10 days	14; *miR-9*, *miR-124a*, *miR-125a*, *miR-125b*, *let-7a*, *let-7b*, *miR-7*, *miR-22*, *miR-23a*, *miR-24*, *miR-26a*, *miR-30a-5p*, *miR-100* and *miR-103; most significant: miR-124a*	–
Evangelisti et al. (2009) [21] Italy	SH-SY5Y	10 μmol/L for 6 days	*miR-128*	–
Le et al. (2009) [32] United States	SH-SY5Y	10 μmol/L for 5 days	6; *miR-7*, *miR-124a*, *miR-125b*, *miR-199a*, miR-199a*, and *miR-214;* most significant: miR-199a*	–
Beverigde et al. (2009) [33] Australia	SH-SY5Y	10 μmol/L for 6 days	12; *miR-10a, miR-128a, miR-331, miR-124a, miR-409-5p, miR-210, miR-149, miR-9, miR-423, miR-483, miR-208* and *miR-184*; most significant: *miR-10a*.	32; miR-301, miR-19a*, miR-520d*, miR-18a*, miR-106a*, miR-218, miR-20a, miR-137, miR-15b, miR-20b*, miR-25*, miR-130a, miR-29b, miR-19b*, miR-34a, let-7a, miR-195, miR-106b*, miR-199a, miR-92*, miR-138, miR-93*, miR-432, miR-519e, miR-101, miR-518f-526a, miR-21, miR-134, miR-98, miR-370, miR-525 and *miR-91 (aka miR-17-5p*); most significant: *miR-301*.
Ragusa et al. (2010) [34] Italy	SK-N-BE	10 μmol/L for 10 days	*3; miR-152, miR-200b* and *miR-338;* most significant: *miR-200b*	–
Meseguer et al. (2010) [35] Spain	SH-SY5Y	1 μmol/L for 4 days	26; miR-10a, miR-10b, miR-10b, miR-615-5p, miR-211, miR-212, miR-132, miR-22, miR-766, miR-135a, miR-148b*, miR-877, miR-184, miR-135b, miR-30e, miR-214, miR-92b, mir-99b, miR-628-3p, miR-27b, miR-335, miR-25, miR-30a, miR-610, miR-331, 3p, miR-149; most significant: *miR-10a* and *miR-10b*	*16; miR-490-3p, miR-154, miR-26a-2, miR-296-3p, miR-422a, miR-486-3p, miR-378, miR-411, miR-543, miR-801, miR-873, miR-107, miR-744, miR-139-3p, miR-487b* and *miR-106a; most significant: miR-490-3p*
Das et al. (2010) [36] Ireland	SK-N-BE	5 μmol/L for 7 days	*17; miR-132, miR-10a, miR-10b, miR-210, miR-192, miR-184, miR-146b, miR-152, miR-126, miR-615, miR-21, miR-203, miR-214, miR-24, miR-196a, miR-191* and *let-7e;* most significant: *miR-132*	*17; miR-409-5p, miR-656, miR-299-5p, miR-485-5p, miR-424, miR-127, miR-323, miR-339, miR-576, miR-432, miR-433, miR-7, miR-30e-5p, miR-485-3p, miR-379, miR-134* and *miR-487b;* most significant: *miR-432*
Das et al. (2012) [37] Ireland	SK-N-BE and SHSY-5Y	5 μmol/L for 7 days	*miR-340*	*–*
Chen et al. (2010) [38] United Kingdom	SH-SY5Y	10 μmol/L for 48 h	6; *miR-132*, *miR-16*, *miR-27a*, *miR-27b*, *miR-214*, and *miR-197;* most significant: *miR-132.*	6; *miR-133a*, *miR-508-3p*, *miR-7*, *miR-1*, *miR-205*, and *miR-20;* most significant: *miR-20b*
Foley et al. (2011) [39] Ireland	SK-N-BE, SHSY-5Y and LAN-5	5 μmol/L for 7 days	30; *miR-132, miR-10a, miR-10b, miR-210, miR-192, miR-184, miR-146b, miR-152, miR-126, miR-615, miR-21, miR-203, miR-214, miR-24, miR-196a, miR-191, miR-7e, miR-28, miR-95, miR-26b, miR-26a, miR-199a, miR-374, miR-340, miR-125b, miR-125a, miR-7 g, miR-425-5p, miR-190, miR- 361*; most significant: *miR-132*, *miR-10a*, and *miR-10b*	23; *miR-409-5p, miR-656, miR-299-5p, miR-485-5p, miR-424, miR-127, miR-323, miR-339, miR-576, miR-432, miR-433, miR-7, miR-30e-5p, miR-485-3p, miR-379, miR-134, miR-487b, miR-382, miR-486, miR-565, miR-149, miR-550* and *miR-503*; most significant: *miR-40*9-5p
Das and Bhattacharyya (2014) [22] (India)	SH-SY5Y	10 μmol/L for 7 days	31; miR-9, miR-9*, miR-15b, miR-16, miR-34a, miR-100, miR-124, miR-125b, miR-126, miR-132, miR-134, miR-137, let-7a, miR-432, miR-15a, miR-21, miR-23a, miR-27b, miR-29c, miR-103, miR-125a, miR-128a, miR-140, miR-141, miR-185, miR-326, miR-1320, let-b, let-7e, miR-127 and *miR-10a*; most significant: *miR-432*	33; *miR-17, miR-19a, miR-138, miR-145, miR-146a, miR-148a, miR-150, miR-154, miR-190 miR-194, miR-200a, miR-205, miR-221, miR-299, miR-323, miR-335, miR-30e, miR-133a, miR-133b, miR-142-5p, miR-144, miR-152, miR-185, miR-189, miR-191, miR-193, miR-200c, miR-213, miR-219, miR-296, miR-302a, miR-331* and *miR-342;* most significant: *miR-17*

*Cluster 17

**Table 2:** miRNAs regulated by ATRA in AML cell lines

Study (reference and country)	Cell type	ATRA treatment	miRNAs upregulated	miRNAs downregulated
Garzon et al. (2007) [23] United States	NB4	10^−1^ μmol /L for 4 days	*9; miR-15a*, *miR-15b*, *miR-16-1*, *let-7a-3*, *let-7c*, *let-7d*, *miR-223*, *miR-342*, and *miR-107; most significant: miR-15b*	*miR-181b*
Marchis et al. (2009) [40] Italy	NB4	1 μmol/L for 72 h	*miR-342*	*–*
Gao et al. (2011) [41] China	U937, HL60, NB4 and leukemic fresh cell lines	1 μmol/L for 72 h	*2; miR-15a* and *miR-16-1*^*;*^ *most significant: miR-15a*	–
Lin et al. (2011) [42] China	NB4, HL60 and K562	1 μmol/L for 72 h	*–*	*miR-125b*
Morris et al. (2013) [43] United States	HL60, NB4, PL21, and THP-1	10^−3^ μmol/L to 1 μmol/L for 21 days	–	*miR-150*
Zhuang et al. (2014) [44] China	NB4	1 μmol/L for 36 h	–	*miR-181a*
Lin et al. (2015) [45] China	HL60 and NB4	3 μmol/L ATRA for 72 h (HL60) and 2 μmol/L ATRA for 4 days (NB4)	*miR-638*	*–*
Bräuer-Hartmann et al. (2015) [46] Germany	NB4	10^−1^ μmol/L for 24 h	–	*miR-181 cluster (miR-181a, miR-181b, miR-181c and miR-181d); most significant: miR-181a* and *miR-181b*
Yan et al. (2016) [47] United States	HL60	10^− 1^ and 1 μmol/L for 72 h	*–*	*miR-17–92* cluster (*miR-17, miR-18a, miR-19b, miR-20a* and *miR-92*); most significant: *miR-20a*

**Table 3:** miRNAs regulated by ATRA in breast carcinoma cell lines

Study (reference and country)	Cell type	ATRA treatment	miRNAs upregulated	miRNAs downregulated
Terao et al. (2011) [48]	MCF-7	1 μmol/L for 72 h	*miR-21*	*–*
Khan et al. (2015) [49] Ireland	T47D and SK-BR-3	1 and 5 μmol/L for 24 h	*miR-10a*	*–*
Fisher et al. (2015) [50] Italy	SKBR3, MCF-7, MDA-MB-157	10^−1^ μmol/L (SKBR3) and 1 μmol/L ATRA (MCF-7 and MDA-MB-157) for 36 h	*13; mir-103*^*1*^*; miR-1225-5p*^*1*^*, miR-1268*^*1*^*, miR-141, miR-188-5p*^*1*^*, miR-19b*^*1*^*, miR-200c*^*1*^*, miR-21*^*1*^*, miR-22*^*1*^*, miR-513a-5p*^*1*^*, miR-575*^*1*^*, miR-642*^*1*^ and *miR-664*^*1a*^	17*; miR-15b*^*12*^*, miR-96*^*12*^*, miR-203*^*12*^*, miR-183*^*12*^*, miR-210-3p*^*123*^*, miR-362-5p*^*12*^*, miR-149*^*12*^*, miR-125a-5p*^*123*^*, miR-193a-5p*^*12*^*, miR-185*^*12*^*, miR-532-5p*^*12*^*, miR-342-3p*^*12*^*, miR-148a*^*1*^*, miR-375*^*1*^*, miR-193a-3p*^*1*^*, miR-205*^*1*^ *and miR-660*^*1a*^

^1^SKBR3 cell lines; ^2^MCF-7 cell lines; ^3^*MDA-MB-157* cell lines*;* ^*a*^Authors did not mention the miRNAs most sensitive to ATRA treatment

**Table 4:** miRNAs regulated by ATRA in lung cancer cell lines

Study (reference and country)	Cell type	ATRA treatment	miRNAs upregulated	miRNAs downregulated
Zhu et al. (2015) [3] China	A549 and H1299	10 and 100 μmol/L for 24 h (A549); and 100 μmol/L for 72 h (H1299)	8*; miR-594, miR-519b, miR-504, miR-512-3p, miR-363, miR-517a, miR-518a* and *miR95;* most significant: *512-3p*	*5; mir-223, miR-196a, miR-369-3p, miR-146a* and *miR-142-3p*^*a*^
Chu et al. (2016) [4] China	H1299 and A549	10 and 100 μmol/L for 72 h	*miR-512-5p*	*–*

^*a*^Authors did not mention the miRNA most sensitive to ATRA treatment

**Table 5:** miRNAs regulated by ATRA in pancreatic cancer, glioma, glioblastoma, embryonal carcinoma, and colorectal cancer

Study (reference and country)	Cell type	ATRA treatment	miRNAs upregulated	miRNAs downregulated
Weiss et al. (2009) [51] Germany	PaTu8988-S and PaTu8988-	1 μmol/L for 72 h	*miR-10a*	*–*
Xia et al. (2009) [52] China	U343 and U251	1 μmol/L for 48 h	*–*	*miR-125b*
Chen et al. [53] (2014) Taiwan	U87 MG	10, 20, 40, and 60 μmol/L for 72 h	*13; miR-302b, miR-302a, miR-302d, miR-30a, miR-146a, miR-224, miR-135a, miR-137, miR-212, miR-628-3p, miR-200c, miR-146b-5p* and *miR-136*; most significant: *miR-302b*	*15; miR-19a, miR-19b, miR-551b, miR-101, miR-301a, miR-199b-5p, miR-455-5p, miR-565, miR-107, miR-216a, miR-32, miR-33a, miR-17-5p, miR-497* and *miR-210;* most significant: *miR-19a*
Chen et al. [54] (2014) China	NT2/D1	10 μmol/L for 21 days	*–*	*miR-134*
Liu et al. [55] (2018) China	HCT116	10, 20, 40, and 60 μmol/L for 24 h	*miR-3666*	*–*

### Neuroblastoma (NB)

NB originates from the aberrant development of primordial neural crest cells and is the most common extracranial solid tumor during childhood and the most common tumor in infants [35]. Several lines of evidence support a role for miRNAs in NB pathogenesis as well as the utility of miRNA profiling in NB diagnostics, classification, and prognosis [35]. Furthermore, the involvement of miRNAs in ATRA-induced differentiation of NB cells was recently reported [35]. In particular, cell lines derived from NB such as SK-N-BE, SH-SY5Y, and LAN-5 can be induced by ATRA treatment to undergo neural cell differentiation and are thus often used as model systems for studying biochemical pathways involved in differentiation [39].

To test the effects of ATRA on NB differentiation and the development of embryonic cells, Chen and Stallings [31] evaluated the expression of 34 human miRNAs in SK-N-BE cells treated with 5 μmol/L ATRA by replacing the culture medium every 24 h for 5 days. The expression profiles of 21 miRNAs were found altered when compared with that of untreated cells (Table 1). Of these miRNAs, 17 were upregulated in ATRA-treated cells and 4 were downregulated. In particular, *miR-184* showed the most significant change, as its expression increased 9-fold following ATRA treatment. The authors also observed that the effects of ATRA treatment on miRNA expression were sustained for at least a short period of time after release; for example, after SK-N-BE cells were treated with ATRA for 5 days and released for 3 days, *miR-184* levels were 25-fold higher than that of untreated cells, indicating sustained effects of ATRA on miRNA expression. In addition, their results suggested that treatment with ATRA is associated with apoptosis rather than differentiation induction in this cell line.

Laneve et al. [20] analyzed the expression pattern of 70 miRNAs in SK-N-BE cells treated with 10 μmol/L ATRA for 3, 6 and 10 days. They found that 14 miRNAs were upregulated (Table 1), 33 did not exhibit any changes in expression, and 23 could not be detected. Expression levels of the upregulated miRNAs were mostly induced after 3 days upon ATRA treatment and progressively increased after terminal differentiation (10 days). Moreover, the authors observed that the expression levels of *miR-9, miR-125b*, and *miR-125a* increased 1.7, 2.2, and 2.6-fold, respectively, compared with that of control cells, and that this increase led to a marked decrease in NB cell proliferation in vitro.

Evangelisti et al. [21] used the same ATRA concentration mentioned above and measured *miR-128* expression in SH-SY5Y cell lines after ATRA treatment. The cells were fed every 48 h with ATRA and then treatment was stopped after 6 days. *miR-128* expression was found upregulated by approximately 3-fold in treated cells compared with untreated ones.

Similarly, Le et al. [32] treated SH-SY5Y cells with 10 μmol/L ATRA but over the course of 5 days. They analyzed the expression profiles of 175 human miRNAs and found that 12 miRNAs were significantly upregulated (miR-106, let-7b, miR-199a*, miR-124a, miR-143, miR-125b, miR-7, miR-189, miR-199a, miR-27a, miR-21, and *miR-214*) during treatment; however, after validation with Northern blot analysis, only six were found upregulated during differentiation (Table 1).

Beverigde et al. [33] treated SH-SY5Y cells with 10 μmol/L ATRA for an additional 1 day longer than Le et al. [32]. They performed microarrays and RT-qPCR to examine miRNA expression profiles. Microarray assays identified 44 miRNAs with altered expression after treatment, of which 12 were significantly upregulated and 32 downregulated (Table 1); however, only 10 miRNAs were confirmed altered by RT-qPCR, of which 3 were upregulated (*miR-128a, miR-10a*, and *miR-124a*) and 7 downregulated *(miR-301, miR-20a, miR-106a, miR-19a, miR-29b, miR-134*, and *miR-15b)*. Interestingly, *miR-9* exhibited a different expression pattern depending on the method of analysis. It was found upregulated via microarray analysis and downregulated via RT-qPCR. ATRA also induced the downregulation of the entire *miR-17* cluster (microarray results). Moreover, five miRNAs that exhibited increased expression (*miR-9*, *miR-124a*, *miR-128a*, *miR-208*, *miR-210*, and *miR-423*) were previously demonstrated to be brain-specific or brain-enriched miRNAs and are considered to play important roles in brain development, neuronal maturation, and neuronal differentiation.

Ragusa et al. [34] analyzed the expression profiles of three miRNAs (*miR-152*, *miR-200b*, and *miR-338*) after ATRA treatment. SK-N-BE cells were treated with ATRA 10 μmol/L and observed on day 5 and 10 after treatment. After 10 days of treatment, all three miRNAs analyzed were upregulated, with the most significant increase observed for *miR-200b*.

In keeping with the above findings, Meseguer et al. [35] showed that ATRA treatment of SH-SY5Y cells resulted in profound changes in miRNA expression patterns. The authors treated SH-SY5Y cells with 1 μmol/L ATRA and assessed changes in miRNA expression at 0, 24, 48, and 96 h. The expression levels of 42 miRNAs were significantly changed [26 upregulated and 16 downregulated (Table 1)]; in particular, *miR-10a* and *10b* showed the most prominent changes in expression. Furthermore, these changes induced by ATRA contributed to the regulation of SH-SY5Y NB cell differentiation and the associated changes in migratory and invasive activities.

Das et al. [36] treated SK-N-BE NB cells with 5 μmol/L ATRA by replacing the culture medium every 24 h for 7 days to determine changes in methylation patterns and gene expression. In addition, they investigated whether upregulated miRNAs are causally associated with the downregulation of a gene known to cause genome-wide demethylation events. They performed expression analysis of 368 miRNAs using low-density TaqMan arrays and found 17 upregulated miRNAs (≥ 2-fold increase) and 17 downregulated miRNAs (≥ 2-fold decrease; Table 1). Among the upregulated miRNAs possibly involved in controlling DNA methylation, ectopic overexpression of *miR-152* significantly decreased cell invasiveness and anchorage independent growth, contributing in part to ATRA-induced differentiation. *miR-152* expression was also analyzed in three other NB cell lines (SH-SY5Y, LAN-5 and SK-N-AS) that received the same treatment. As observed in SK-N-BE cells, ATRA treatment induced *miR-152* upregulation in SH-SY5Y and LAN-5 cell lines; however, this miRNA was downregulated in SK-N-AS cells.

In a subsequent study with the same treatment regimen, Das et al. [37] observed a correlation between changes in miRNA expression profiles and methylation following ATRA treatment. In SK-N-BE cells, 20 miRNAs were found upregulated and 24 downregulated following ATRA treatment, whereas 13 miRNAs were upregulated in SH-SY5Y cells (the authors did not specify which miRNAs are altered). Only *miR-340* was upregulated in both cell lines and showed the highest expression levels. In addition, overexpression of *miR-340* was associated with decreased cell viability and limited colony-forming ability.

Chen et al. [38] observed significant changes in the expression profiles of 12 miRNAs, of which half were upregulated and half downregulated, when SH-SY5Y cells were treated with 10 μmol/L ATRA for 48 h (Table 1). Foley et al. [39] treated NB cell lines (SK-N-BE, SH-SY5Y, and LAN-5) with 5 μmol/L ATRA by exchanging the culture medium every 24 h for 7 days, and then analyzed the expression profiles of 364 miRNAs. In SK-N-BE cells, several miRNAs exhibited altered expression levels, of which 53 miRNAs showed significant changes—30 were positively regulated and 23 negatively regulated (Table 1). miRNAs with the highest changes in expression (410-fold increase by day 7) included *miR-132*, *miR-10a*, and *miR-10b*. Similarly, these miRNAs were significantly upregulated in response to ATRA in SK-N-BE, SHSY-5Y, and LAN-5 NB cells.

Das and Bhattacharyya [22] treated SH-SY5Y cells for 7 days with 10 μmol/L ATRA and investigated the expression levels of 96 miRNAs on day 3 and 7. They found 31 upregulated miRNAs, of which 14 exhibited high expression levels (miR-9, miR-9 *, miR-15b, miR-16, miR-34a, miR-100, miR-124, miR-125b, miR-126, miR-132, miR-134, miR-137, let-7a, and *miR-432*), and 33 downregulated miRNAs (Table 1). Interestingly, *miR-185* presented with two different patterns of expression, downregulation on day 3 and upregulation on day 7.

These results support the notion that miRNA regulation plays a key role in the differentiation of NB cells induced by ATRA and in the phenotypic changes linked to the expression of genes associated with these miRNAs. In addition, multiple lines of evidence indicate that NB treated with ATRA exhibits lower migration and invasion abilities. Changes in miRNA expression profiles in NB cell lines after ATRA treatment are summarized in Table 1**.**

### Acute myeloid leukemia (AML)

AML is characterized by abnormal differentiation and uncontrolled proliferation of immature hematopoietic cells [56]. In hematopoietic malignancies, several miRNAs have been reported to exhibit tumor-suppressive or oncogenic roles in leukemogenesis [44]. Accordingly, it has been suggested that miRNAs are important in the molecular pathogenesis of leukemia by interfering with essential pathways of hematopoietic differentiation [40].

Acute promyelocytic leukemia (APL), the main subtype of acute myeloid leukemia in which positive responses are observed after treatment with ATRA [56], is characterized by chromosomal translocations involving the RA receptor-α (*RARA*) gene that result in clonal expansion of hematopoietic precursors blocked at the promyelocytic stage of differentiation [40]. Several miRNAs upregulated upon ATRA treatment of APL cell lines have already been identified in different studies [40].

Garzon et al. [23] cultured APL NB4 cells with or without 10^− 1^ μmol ATRA over 4 days and identified nine upregulated and one downregulated miRNA after comparing expression levels between both groups (Table 2). They also observed that treatment of NB4 cells with ATRA induced granulocytic differentiation, as evidenced by morphological changes and increased expression of the surface antigens CD11b and CD15.

Marchis et al. [40] treated NB4 APL cells with 1 μmol/L ATRA and confirmed that ATRA induced a marked and selective increase in *miR-342* expression, starting 24 h after treatment and progressively increasing over 3 days. They also found increased expression of the *miR-342* host gene Enah/Vasp-like (*EVL*) during ATRA treatment. These results indicated that *EVL* and *miR-342* expression is co-regulated, with *miR-342* contributing to the ATRA-mediated granulocytic differentiation program of promyelocytic precursors in ATRA-treated NB4 cells.

To uncover the regulation of *miR-15a* and *miR-16-1* induced by ATRA, Gao et al. [41] employed two models, NB4, HL60, and U937 cell lines and fresh leukemic cells extracted from 10 AML patients; both experimental underwent 1 μmol/L ATRA treatment for 72 h. The authors found that treatment with ATRA increased *miR-15a* and *miR-16-1* expression in NB4 cells, which in turn culminated in the inhibition of leukemic cell differentiation. They also observed that *miR-15a* and *miR-16-1* were upregulated in 8 of 10 patient samples. These findings indicated that upregulation of *miR-15a* and *miR-16-1* may be associated with the differentiation induced by ATRA. In addition, they evaluated the time (0 to 72 h) and concentration-dependent (10^− 2^ to 10 μmol/L ATRA) expression of *miR-15a* and *miR-16-1* in response to ATRA treatment, which induced differentiation in NB4 cells. They found that an increase in time and ATRA concentrations between 10^− 1^ to 10 μmol/L ATRA were associated with elevated *miR-15a* and *miR-16-1* expression; however, 10^− 2^ μmol/L ATRA did not induce differentiation and failed to modulate the expression of *miR-15a and miR-16-1.*

In another study, Lin et al. [42] treated NB4, HL60, and K562 cells with 1 μmol/L ATRA over 72 h to monitor the in vitro expression levels of *CDX2* and *miR-125b*. In NB4 cells, *miR-125b* expression progressively decreased to 30% of baseline after 72 h following differentiation induction with ATRA. The researchers also demonstrated decreased *CDX2* mRNA expression levels that were positively correlated with *miR-125b* levels after ATRA treatment, suggesting a potential function of *CDX2* and *miR-125b* in inhibiting cell differentiation in AML cells and promoting leukemogenesis.

Morris et al. [43] further examined AML cell lines (NB4, HL60, PL21, and THP-1) that can be induced to differentiate along the granulocytic or monocytic lineage after exposure to ATRA (10^− 3^ to 1 μmol/L) for 2 (THP-1), 3 (NB4) or 4 (HL60 and PL21) days. The authors found that these AML cells lines express very low levels of endogenous *miR-150* in contrast to normal CD34+ progenitors cell lines after exposure to ATRA. However, in AML cell lines, differentiation of *miR-150*-expressing cells occurs independently of RARA signaling.

Zhuang et al. [44] revealed the downregulation of *miR-181a* in NB4 cells during treatment with 1 μmol/L ATRA over 36 h. They also observed that ATRA induced NB4 cell differentiation. Their study strongly supported the therapeutic role of ATRA on APL. Furthermore, Lin et al. [45] observed significant upregulation of *miR-638* during ATRA-induced myeloid differentiation of HL60 and NB4 cell lines compared with that of untreated control. HL60 cells were treated with 3 μmol/L ATRA for 72 h while NB4 cells were treated with 2 μmol/L ATRA for 4 days. In HL60 cells, upregulation of *miR-638* coincided with increased expression of the myeloid-specific surface markers CD14 and CD11b. Moreover, CDK2 overexpression eliminated the inhibitory effect of *miR-638* in HL60 cells, which was more significant upon ATRA treatment.

To evaluate changes in miRNA expression mediated by ATRA, Bräuer-Hartmann et al. [46] cultured NB4 cell lines in the presence of 10^− 1^ μmol/L ATRA and analyzed the expression profiles of *miR-181* family members (*miR-181a–d*) 24 h after treatment. In this study, a significant downregulation of all *miR-181* family members was observed. In parallel, they also investigated granulocyte differentiation associated with changes in the expression patterns of the *miR-181* family in U937, HL60, and NB4 cells treated with 1 μmol/L ATRA. They found significant downregulation of *miR-181a/b* in APL cell lines (NB4); however, no significant changes were found in the non-APL cell lines (U937 and HL60). Moreover, inhibition of the *miR-181a/b* cluster by ATRA treatment effectively repressed cell proliferation and induced apoptosis in APL cells (NB4).

After treatment of AML HL60 cells with 0.1 or 1 μmol/L ATRA for 72 h, Yan et al. [47] found a significant reduction in the expression levels of the *miR-17–92* cluster upon ATRA treatment, which decreased by almost 50% in 3 days. In addition, they found that HL60 cell proliferation was reduced after exposure to ATRA, which activated their terminal differentiation into granulocytes.

In summary, these studies demonstrated that several miRNAs are highly expressed in specific hematopoietic cell lineages and that modulation of their expression induced by ATRA treatment may be correlated with changes in cellular properties or differentiation and may thus represent an effective treatment strategy. miRNAs induced in AML cell lines after ATRA treatment are summarized in Table 2.

### Breast carcinoma

Breast cancer is the most common malignancy in women [57] and represents a heterogeneous group of tumors with varying responses to therapeutic agents, including retinoids [48]. Therefore, only a few reports have assessed the effects of retinoids on miRNAs in this carcinoma.

For example, Terao et al. [48] determined the differential profiles of miRNA expression in MCF-7 and MDA-MB-231 cells cultured with or without 1 μmol/L ATRA over 72 h. Although ATRA did not affect the miRNA profiles of MDA-MB-231 cells—as they are estrogen receptor-negative (ERα-)—the retinoid significantly increased expression levels of a single miRNA, *miR-21*, in MCF-7 cells (estrogen receptor-positive; ERα+) after 2 h, which leveled off at 24 h.

To investigate the potential modulation of *miR-10a* expression in breast cancer cells, Khan et al. [49] analyzed SKBR3 cells exposed to higher ATRA concentrations (1 and 5 μmol/L)—albeit for shorter duration (24 h). For both concentrations, an increase in *miR-10a* expression was observed, with higher expression levels induced upon exposure to 5 μmol/L ATRA. The authors also analyzed T47D cells exposed to 1 or 5 μmol/L ATRA for 24 h and found that *miR-10a* expression was similarly upregulated upon stimulation under both ATRA concentrations.

Fisher et al. [50] treated SKBR3 cells with 10^− 1^ μmol/L ATRA for up to 36 h and then observed the expression profiles of 57 miRNAs, of which 37 were upregulated and 20 downregulated. The authors chose to only analyze 30 of these miRNAs in detail (Table 3), because they are strongly associated with positive or negative regulation of more than 500 mRNAs involved in different stages of tumor progression (e.g., apoptosis, motility, and cell invasion). This indicates a possible role for ATRA in the control of the metastatic invasiveness of breast cancer. In addition, the authors treated MCF-7, MDA-MB-453, MDA-MB-231, and MDA-MB-157 cells with 1 μmol/L ATRA for up to 36 h. In MCF-7 cells, ATRA negatively regulated 12 miRNAs, which were also downregulated in SKBR3 cells. They also observed a significant ATRA-dependent downregulation of *miR-125a-5p* and *miR-210-3p* in MDA-MB-157 cells. On the other hand, none of the miRNAs were found modulated in response to ATRA in MDA-MB-231 and MDA-MB-453 cells. miRNA expression profiles in breast carcinoma cell lines after ATRA treatment are summarized in Table 3.

### Lung cancer

Lung cancer is responsible for the largest number of deaths among all cancer types in both men and women [3]. Recently, it has been demonstrated that miRNAs are directly involved in lung cancer development and progression [3].

Zhu et al. [3] treated non-small cell lung cancer A549 cells with 10 or 100 μmol/L ATRA for 12 or 24 h and H1299 cells with 100 μmol/L ATRA for 48 or 72 h. The authors observed changes in the expression profiles of 13 miRNAs in A549 cell lines (Table 4); however, they focused on *miR-512-3p* because it exhibited a significant response to ATRA treatment and pro-apoptotic tumor suppressor abilities. In A549 cells, *miR-512-3p* expression increased by approximately 100% at 12 h and reached a peak at 24 h upon 100 μmol/L ATRA treatment, whereas expression in H1299 cells increased by only 7-fold after 72-h treatment with 100 μmol/L ATRA and showed no change at 48 h. In addition, they demonstrated that *miR-512-3p* overexpression inhibited adhesion, migration, and invasion of A549 and H1299 lung cancer cells.

To explore the effects of RA on the expression profile of *miR-512-p*, Chu et al. [4] also treated H1299 and A549 cells with 10 or 100 μmol/L ATRA for 24, 48, and 72 h. miRNA expression levels in A549 cells increased 3-fold when exposed to 10 μmol/L ATRA across all time points compared with that of control. When ATRA concentration increased to 100 μmol/L, *miR-512-5p* expression increased by 5, 17, and 3-fold at 24, 48, and 72 h, respectively. In H1299 cells, *miR-512-5p* expression increased by approximately 4-fold and 10-fold when treated with 10 μmol/L and 100 μmol/L ATRA, respectively, at all three time points compared with that of control. They also found that apoptosis was stimulated in both lung cancer cell lines. Overexpression of *miR-512-5p* in A549 and H1299 cells led to a 40 and 46% increase, respectively, in apoptotic cells compared with that of control cells. In addition, ATRA treatment and the consequent increase in *miR-512-p* expression led to a sharp decrease in glucose uptake by tumor cells and attenuated cell migration capacity by 39% compared to that of control. miRNA expression profiles of lung cancer cell lines after ATRA treatment are summarized in Table 4.

### Other neoplasms

Weiss et al. [51] investigated the importance of *miR-10a* in pancreatic cancer metastasis and the possible interactions between *miR-10a* expression and RA. The authors employed the pancreatic tumor cell lines PaTu8988-S and PaTu8988-T and administered 1 μmol/L ATRA or a selective RARα antagonist for 72 h. They found that *miR-10a* expression in nonmetastatic pancreatic cells was positively regulated by ATRA stimulation, whereas *miR-10a* expression significantly decreased in cells treated with a selective RARα antagonist.

In another study, Xia et al. [52] treated U343 and U251 glioma cell lines with 1 μmol/L ATRA for 48 h and found that ATRA treatment negatively regulates *miR-125b* expression in both cell lines, although U343 cells were more sensitive to ATRA than were U251 cells. Furthermore, ATRA-mediated *miR-125b* overexpression led to reduced cell proliferation and increased apoptosis in both cell lines.

Human glioblastoma cells (U87 MG) were administered different doses of ATRA (10, 20, 40, and 60 μmol/L) and analyzed after 24, 48 and 72 h in the study by Chen et al. [53]. ATRA was found to positively regulate different members of the *miR-302* cluster (Table 5), especially *miR-302b*, in a dose-dependent manner. Treatment with 40 μmol/L ATRA more than doubled *miR-302b* expression 12 h after exposure, and this overexpression was positively associated with cell death, suggesting that an increase in *miR-302b* expression is associated with suppression of tumorigenesis. Moreover, cell death associated with ATRA treatment may be regulated via *miR-320b* expression.

In another study by Chen et al. [54], pluripotent human embryonal carcinoma cells (NT2/D1) were treated with 10 μmol/L ATRA for 21 days. A decrease in the expression levels of the transcription factor FOXM1 was observed, which was mediated by the ATRA-induced overexpression of *miR-134*. Moreover, *miR-134* overexpression resulted in decreased NT2/D1 cell pluripotency. ATRA was found to function by binding to the ligand-inducible transcription factors that activate or repress the transcription of downstream target genes, controlling cell growth and differentiation in both embryonic and adult cells.

Liu et al. [55] further investigated the association between *miR-3666* expression and the effects of ATRA treatment on human colorectal cancer (CRC) cells. After HCT116 cells were treated with increasing doses of ATRA (10, 20, 40, and 60 μmol/L) for 24 h, ATRA concentrations between 20 and 60 μmol/L were found to increase *miR-3666* expression in a dose-dependent manner. Moreover, ATRA-induced positive modulation of miR-3666 in HCT116 cells was associated with regulation of cancer cell viability, apoptosis, migration, and invasiveness. The authors also reported that 40 μmol/L ATRA treatment can decrease CRC cell viability by 50%, which was mediated by *miR-3666* expression. Thus, ATRA may be a potential preventative agent against CRC development by regulating *miR-3666* expression.

## Discussion

The studies included in this review demonstrated that more than 300 miRNAs are induced upon ATRA treatment in neoplastic cell lines. The ATRA concentrations used in these studies varied greatly (10^− 3^ μmol/L to 10^2^ μmol/L). In addition, the duration of ATRA treatment ranged from 1 to 21 days, with the most common duration being 3 days. Overall, the findings indicated that the optimum ATRA treatment parameters to determine changes in miRNA expression in neoplastic cell lines are 10 μmol/L and 3 days.

The findings demonstrated that *miR-10a* is possibly the most sensitive to ATRA treatment, considering that this miRNA showed altered expression patterns in response to ATRA in seven different studies [33, 35, 36, 39, 49, 51], which addressed three different types of cancers—neuroblastoma, breast cancer, and pancreatic cancer. In addition, treatment of NB cell lines with 5 μmol/L ATRA for 7 days led to a 410-fold increase in *miR-10a* expression. Moreover, *miR-10a* was the most common miRNA among all upregulated miRNAs, as it was found upregulated in all studies that measured its expression. Meanwhile, *miR-134* was the most common downregulated miRNA [33, 36, 39, 54] and was associated with two different types of cancer, neuroblastoma and embryonal carcinoma.

We observed a large variation in the number of miRNAs evaluated in each study, with some studies analyzing more than 300 miRNAs and others only a single miRNA. In addition, some authors chose not to elucidate in depth all miRNAs that were sensitive to ATRA treatment or the significant findings associated with ATRA exposure, which may have led to imprecise conclusions. Finally, several studies demonstrated that ATRA largely inhibits cell growth and proliferation and promotes apoptosis of neoplastic cells.

## Conclusion

Overall, our systematic review supports the use of ATRA as a potential strategy for the prevention and treatment of various neoplasms. However, it should be noted that these studies may not accurately represent changes occurring in vivo, which highlights the need for additional studies to fully elucidate the modulation of miRNAs induced by ATRA in the treatment of neoplasms.

## Data Availability

Not applicable.

## Abbreviations

AML: acute myeloid leukemia
APL: acute promyelocytic leukemia
ATRA: all-trans retinoic acid
CRC: colorectal cancer
ERα-: estrogen receptor-negative
ERα+: estrogen receptor-positive
EVL: Enah/Vasp-like
miRNAs: microRNAS
NB: neuroblastoma
RA: retinoic acid
RARA: retinoic acid receptor-α
